# Supplementary material for: Smooth Interpolating Curves with Local Control and Monotone Alternating Curvature
Source: Comput Graph Forum. 2022 Oct 6;41(5):25–38. doi: 10.1111/cgf.14600 (PMC9827861; doi:10.1111/cgf.14600)
Supplement: Supplementary file 1 — Supplement Material [file CGF-41-25-s001.zip › Local-Smooth-Interpolating-MonoCurvature/extern/clothoids/submodules/Utils/docs/api-cpp/program_listing_file_Utils.cc.html]

Program Listing for File Utils.cc — Utils v1.2

### Navigation

- index
- toc
- Utils »
- Program Listing for File Utils.cc

# Program Listing for File Utils.cc¶

↰ Return to documentation for file (`Utils.cc`)

```
/*--------------------------------------------------------------------------*\
 |                                                                          |
 |  Copyright (C) 2017                                                      |
 |                                                                          |
 |         , __                 , __                                        |
 |        /|/  \               /|/  \                                       |
 |         | __/ _   ,_         | __/ _   ,_                                |
 |         |   \|/  /  |  |   | |   \|/  /  |  |   |                        |
 |         |(__/|__/   |_/ \_/|/|(__/|__/   |_/ \_/|/                       |
 |                           /|                   /|                        |
 |                           \|                   \|                        |
 |                                                                          |
 |      Enrico Bertolazzi                                                   |
 |      Dipartimento di Ingegneria Industriale                              |
 |      Universita` degli Studi di Trento                                   |
 |      email: enrico.bertolazzi@unitn.it                                   |
 |                                                                          |
\*--------------------------------------------------------------------------*/

#ifndef DOXYGEN_SHOULD_SKIP_THIS

#include "Utils.hh"

namespace Utils {

  using std::string;
  using std::lower_bound;

  /*\
  :|:  _
  :|: | |__  __ _ ___ ___ _ _  __ _ _ __  ___
  :|: | '_ \/ _` (_-</ -_) ' \/ _` | '  \/ -_)
  :|: |_.__/\__,_/__/\___|_||_\__,_|_|_|_\___|
  :|:
  \*/

  #ifdef UTILS_OS_WINDOWS
    string
    basename( char const * const path ) {
      static char drive[100];
      static char dir[1024];
      static char fname[256];
      static char ext[128];
      errno_t e = _splitpath_s(
        path,
        drive, 100,
        dir,   1024,
        fname, 256,
        ext,   128
      );
      UTILS_ASSERT0( e == 0, "lapack_wrapper, basename failed!\n" );
      return fname;
    }
  #else
    string
    basename( char const * const path ) {

      if ( path[0] == '\0' ) return string("");

      string filename(path);

      size_t len   = filename.length();
      size_t index = filename.find_last_of("/\\");

      if ( index == string::npos ) return filename;
      if ( index + 1 >= len ) {
        --len;
        index = filename.substr(0, len).find_last_of("/\\");

        if ( len   == 0 ) return filename;
        if ( index == 0 ) return filename.substr(1, len - 1);
        if ( index == string::npos ) return filename.substr(0, len);
        return filename.substr(index + 1, len - index - 1);
      }
      return filename.substr(index + 1, len - index);
    }
  #endif

  /*\
   |                           _     ___       _                       _
   |   ___  ___  __ _ _ __ ___| |__ |_ _|_ __ | |_ ___ _ ____   ____ _| |
   |  / __|/ _ \/ _` | '__/ __| '_ \ | || '_ \| __/ _ \ '__\ \ / / _` | |
   |  \__ \  __/ (_| | | | (__| | | || || | | | ||  __/ |   \ V / (_| | |
   |  |___/\___|\__,_|_|  \___|_| |_|___|_| |_|\__\___|_|    \_/ \__,_|_|
  \*/

  template <typename T_int, typename T_real>
  void
  searchInterval(
    T_int                npts,
    T_real const * const X,
    T_real             & x,
    T_int              & lastInterval,
    bool                 closed,
    bool                 can_extend
  ) {

    // check points
    T_int n  = npts-1;
    UTILS_ASSERT(
      npts > 1 && lastInterval >= 0 && lastInterval < n,
      "In searchInterval( npts={}, X, x={}, lastInterval={}, closed={}, can_extend={})\n"
      "npts musrt be >= 2 and lastInterval must be in [0,npts-2]\n",
      npts, lastInterval, closed, can_extend
    );

    // checl range
    T_real xl = X[0];
    T_real xr = X[n];
    if ( closed ) { // put x in range (change also its value)
      T_real L = xr-xl;
      x -= xl;
      x  = fmod( x, L );
      if ( x < 0 ) x += L;
      x += xl;
    } else {
      UTILS_ASSERT(
        can_extend || (x >= xl && x <= xr),
        "In searchInterval( npts={}, X, x={}, lastInterval={}, closed={}, can_extend={})\n"
        "out of range: [{},{}]\n",
        npts, lastInterval, closed, can_extend, xl, xr
      );
    }

    // find the interval of the support of the B-spline
    T_real const * XL = X+lastInterval;
    if ( XL[1] < x ) { // x on the right
      if ( x >= X[n-1] ) {
        lastInterval = n-1; // last interval
      } else if ( x < XL[2] ) { // x in (XL[1],XL[2])
        ++lastInterval;
      } else { // x >= XL[2] search the right interval
        T_real const * XE = X+n;
        lastInterval += T_int(lower_bound( XL, XE, x )-XL);
        T_real const * XX = X+lastInterval;
        if ( x < XX[0] || Utils::isZero(XX[0]-XX[1]) ) --lastInterval;
      }
    } else if ( x < XL[0] ) { // on the left
      if ( x <= X[1] ) { // x in [X[0],X[1]]
        lastInterval = 0; // first interval
      } else if ( XL[-1] <= x ) { // x in [XL[-1],XL[0])
        --lastInterval;
      } else {
        lastInterval = T_int(lower_bound( X+1, XL, x )-X);
        T_real const * XX = X+lastInterval;
        if ( x < XX[0] || Utils::isZero(XX[0]-XX[1]) ) --lastInterval;
      }
    } else {
      // x in the interval [ XL[0], XL[1] ] nothing to do
    }
    // check computed interval
    UTILS_ASSERT(
      lastInterval >= 0 && lastInterval < n,
      "In searchInterval( npts={}, X, x={}, lastInterval={}, closed={}, can_extend={})\n"
      "computed lastInterval of range: [{},{}]\n",
      npts, lastInterval, closed, can_extend, xl, xr
    );

  }

  extern template void searchInterval(
    int32_t             npts,
    float const * const X,
    float             & x,
    int32_t           & lastInterval,
    bool                closed,
    bool                can_extend
  );

  template void searchInterval(
    int32_t              npts,
    double const * const X,
    double             & x,
    int32_t            & lastInterval,
    bool                 closed,
    bool                 can_extend
  );

  template void searchInterval(
    int64_t             npts,
    float const * const X,
    float             & x,
    int64_t           & lastInterval,
    bool                closed,
    bool                can_extend
  );

  template void searchInterval(
    int64_t              npts,
    double const * const X,
    double             & x,
    int64_t            & lastInterval,
    bool                 closed,
    bool                 can_extend
  );
}

#endif
```

### Quick search

### Table of Contents

- rang - Colors for your Terminal
- Zstr
- C++ API

«
hide menu

menu
sidebar
»

### Navigation

- index
- toc
- Utils »
- Program Listing for File Utils.cc

© Copyright 2021, Enrico Bertolazzi.
Created using Sphinx 4.2.0.
